# MDE-DETR: multi-domain enhanced feature fusion algorithm for bayberry detection and counting in complex orchards

**DOI:** 10.3389/fpls.2025.1711545

**Published:** 2025-11-27

**Authors:** Cheng Zhou, Yuyu Zhang, Wei Fu, Lili Yao, Chengliang Yin

**Affiliations:** 1 School of Information Engineering, Huzhou University, Huzhou, China; 2 Mechanical and Electrical Engineering College, Hainan University, Haikou, China

**Keywords:** bayberry, object detection, complex environment, multi-scale feature fusion, deeplearning

## Abstract

**Introduction:**

Bayberry detection plays a crucial role in yield prediction. However, bayberry targets in complex orchard environments present significant detection challenges, including small volume, severe occlusion, and dense distribution, making traditional methods inadequate for practical applications.

**Methods:**

This study proposes a Multi-Domain Enhanced DETR (MDE-DETR) detection algorithm based on multi-domain enhanced feature fusion. First, an Enhanced Feature Extraction Network (EFENet) backbone is constructed, which incorporates Multi-Path Feature Enhancement Module (MFEM) and reparameterized convolution techniques to enhance feature perception capabilities while reducing model parameters. Second, a Multi-Domain Feature Fusion Network (MDFFN) architecture is designed, integrating SPDConv spatial pixel rearrangement, Cross-Stage Multi-Kernel Block (CMKBlock), and dual-domain attention mechanisms to achieve multi-scale feature fusion and improve small target detection performance. Third, an Adaptive Deformable Sampling (ADSample) downsampling module is constructed, which dynamically adjusts sampling positions through learnable spatial offset prediction to enhance model robustness for occluded and dense targets.

**Results and discussion:**

Experimental results demonstrate that on a self-constructed bayberry dataset, MDE-DETR achieves improvements of 3.8% and 5.1% in mAP50 and mAP50:95 respectively compared to the RT-DETR baseline model, reaching detection accuracies of 92.9% and 67.9%, while reducing parameters and memory usage by 25.76% and 25.14% respectively. Generalization experiments on VisDrone2019 (a small-target dataset) and TomatoPlantfactoryDataset (a dense occlusion dataset) datasets further validate the algorithm's effectiveness, providing an efficient and lightweight solution for small-target bayberry detection in complex environments.

## 1 Introduction

The agricultural sector faces unprecedented challenges in meeting the growing global food demand while maintaining sustainable production practices. Bayberry, a high-nutritional-value fruit rich in glucose, cellulose, mineral elements, and amino acids, holds significant economic potential in the global fruit market (Liu et al., 2020). However, manual counting of bayberry fruits in complex orchard environments remains a labor-intensive and time-consuming process, limiting the efficiency of yield estimation and automated harvesting systems. Developing intelligent detection algorithms capable of accurately identifying and counting small-target bayberries under challenging conditions such as varying illumination, leaf and branch occlusion, and dense fruit clustering has become increasingly important for advancing precision agriculture and supporting the digital transformation of orchard management practices (Koirala et al., 2019).

Traditional fruit detection methods in orchard environments primarily rely on handcrafted feature extraction methods and classical machine learning techniques. Early approaches employed color-based segmentation techniques, using clustering algorithms such as k-means, statistical feature extraction, and morphological operations to identify fruits in complex backgrounds (Bargoti and Underwood, 2017). However, traditional methods exhibit significant limitations, including high sensitivity to illumination changes, complex background interference, and the need for extensive manual feature engineering, making them insufficient to handle the inherent complexity and variability in real orchard environments (Bresilla et al., 2019).

The emergence of deep learning has revolutionized fruit detection applications, with Convolutional Neural Networks (CNNs) demonstrating superior performance compared to traditional methods. Modern deep learning frameworks have been successfully applied to various fruit detection tasks, achieving remarkable detection accuracy in complex agricultural environments. Mohanty et al. demonstrated the technical feasibility of smartphone-assisted crop disease diagnosis using deep convolutional neural networks, achieving 99.35% accuracy on a dataset of 54,306 images of 14 crop species and 26 diseases (Mohanty et al., 2016). Lawal et al. proposed an improved YOLOv5s model using feature concatenation with attention mechanism for real-time fruit detection, achieving 93.4% mAP with reduced computational costs and 74 fps detection speed (Lawal et al., 2023). Kirk et al. developed the Lab-Fruits detection system, combining bio-inspired color processing methods with single-stage deep learning networks to achieve fast and robust detection of soft fruits such as strawberries in real industrial environments (Kirk et al., 2020).

Given the maturity and effectiveness of modern object detection algorithms in handling complex visual recognition tasks, adopting object detection frameworks has become the preferred approach for fruit detection applications in recent years. Recently, numerous researchers have focused on improving YOLO algorithms (Redmon et al., 2016) to adapt to agricultural detection requirements. Lin et al. developed DPD-YOLO for dense pineapple fruit detection in complex environments, achieving 62.0% mAP@0.5 with 6.6% improvement over original YOLOv8 (Lin et al., 2025). Zhao et al. developed YOLO-Granada, a lightweight attention model specifically for pomegranate fruit detection, achieving a 17.3% speed improvement and significant model compression (Zhao et al., 2024). Wang et al. proposed the NVW-YOLOv8s network, which effectively addressed category imbalance and small target occlusion problems in tomato fruit detection at different maturity stages through foreground category balancing methods and improved detection segmentation algorithms (Wang et al., 2024).

Recent developments in Transformer-based detection models (Vaswani et al., 2017), particularly the introduction of RT-DETR (Lv et al., 2023), have demonstrated tremendous potential for end-to-end object detection without requiring post-processing steps such as Non-Maximum Suppression (NMS). Wang et al. proposed the HESP-DETR model, implementing pomegranate growth stage detection based on improved RT-DETR, significantly improving detection accuracy while maintaining real-time performance through multi-scale multi-head self-attention (MMSA) mechanisms (Wang et al., 2025). Wang et al. developed PDSI-RTDETR, a lightweight tomato maturity detection algorithm that achieved 3.9% mAP50 and 38.7% FPS improvements compared to the original RT-DETR model by integrating PConv partial convolution, deformable attention mechanisms, and SlimNeck-SSFF feature fusion structures (Wang et al., 2024). Wu et al. proposed an improved RT-DETR crop maturity detection method that enhanced detection accuracy while reducing model size and computational complexity (Wu et al., 2025). Jrondi et al. compared the performance of DETR and YOLOv8 in citrus fruit detection, validating the effectiveness of Transformer-based end-to-end detection methods in complex agricultural environments (Jrondi et al., 2024). These studies fully demonstrate the tremendous potential and broad application prospects of RT-DETR in agricultural object detection, leading us to choose RT-DETR as the base architecture for the MDE-DETR model.

Despite significant advances in object detection algorithms, while RT-DETR performs excellently in general object detection tasks, several key challenges remain when applying these methods to small-target bayberry detection in complex orchard environments. The small size and irregular distribution of bayberry fruits, along with severe occlusion, present difficulties for existing detection models, which often have deficiencies in multi-scale feature representation. Complex orchard backgrounds with varying lighting conditions, dense leaf occlusion, and overlapping fruit clusters generate significant interference, reducing detection accuracy.

To address these challenges, we propose MDE-DETR, a novel small-target bayberry detection algorithm tailored for complex orchard environments. The main contributions of this work are as follows:

1. We design an enhanced feature extraction network that integrates Multi-Path Feature Enhancement Module (MFEM) and reparameterized convolution techniques, significantly improving the network’s perception capability for small target features. The EFENet architecture employs cross-stage partial connections and enhanced local attention mechanisms to optimize feature representation and gradient propagation efficiency.
2. We propose a multi-domain feature fusion network that integrates SPDConv spatial pixel reorganization, Cross-Stage Multi-Kernel Block (CMKBlock), and Dual-Domain Attention Module (DDAM) to achieve efficient multi-scale feature fusion. This architecture leverages spatial and frequency domain information to enhance feature discrimination capability while maintaining computational efficiency.
3. We design an adaptive deformable sampling module that utilizes learnable spatial offset prediction mechanisms to dynamically adjust sampling positions during downsampling operations. This method effectively preserves key feature information of small targets while adapting to irregular fruit distributions in complex orchard environments.

## 2 Related work

### 2.1 Dataset construction

**Figure 1:**
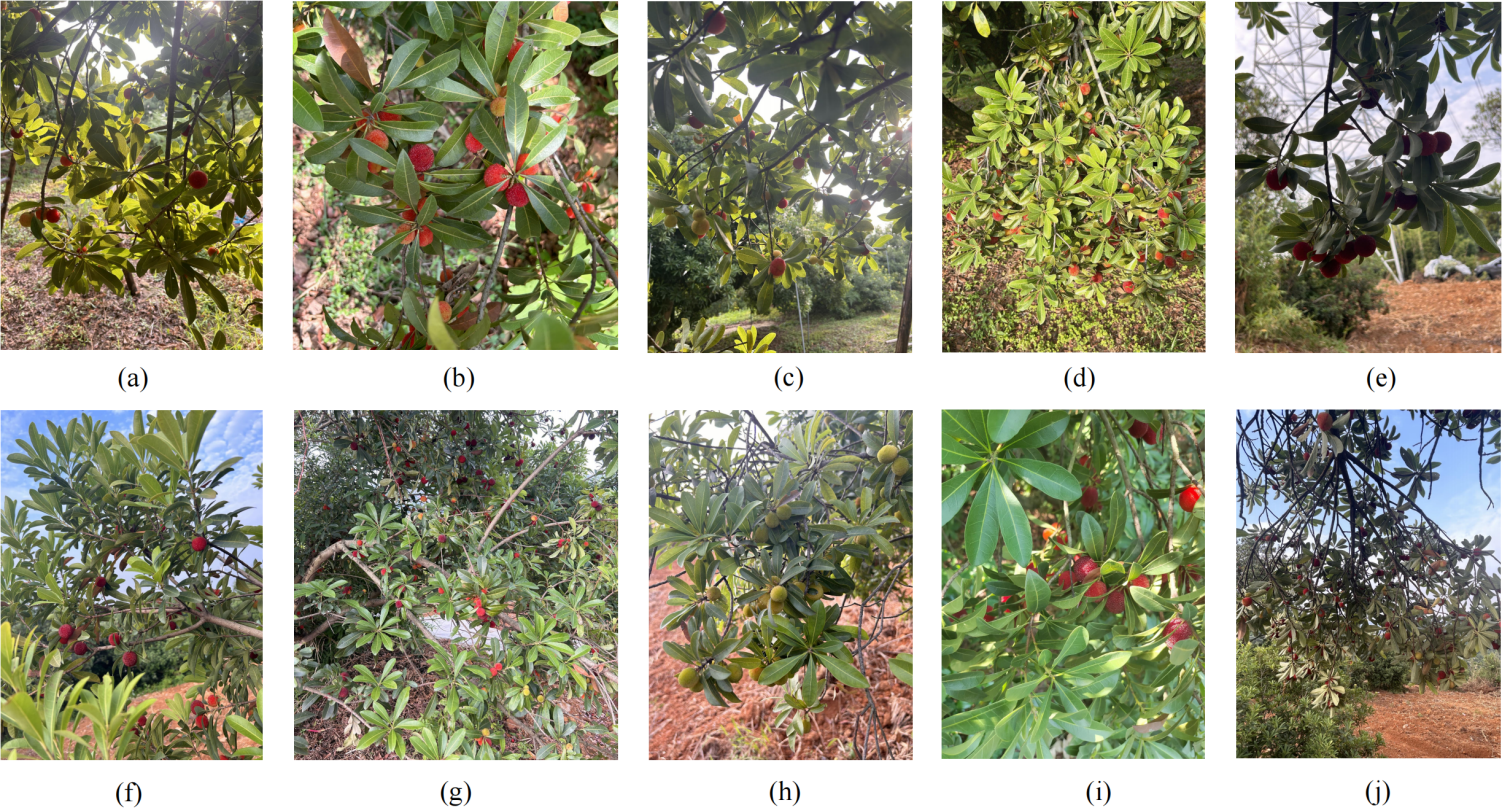
Sample images from bayberry dataset **(a)** backlight; **(b)** frontlight; **(c)** upward angle; **(d)** downward angle; **(e)** close-up; **(f)** medium view; **(g)** distant view; **(h)** green fruit; **(i)** red fruit; **(j)** severe occlusion.

The bayberry data collection for this study was conducted at the Zhushan Bayberry Industry Park in Changxing County, Huzhou City, Zhejiang Province, spanning from early May to late June, covering the main growing season of bayberries. During the collection process, we used smartphones including iPhone 12, iPhone 14, and Redmi K40S for photography. To increase data diversity, we captured images under different lighting conditions. We also employed diverse shooting angles, including close-up, medium, and distant views, as well as different elevation and depression angles, to ensure the model could adapt to various shooting conditions. Special attention was paid to areas with dense fruit distribution on bayberry trees, while also including fruits obscured by leaves to improve model robustness. Finally, we collected 2,512 original images, providing sufficient material for subsequent data processing and model training. Some sample images are shown in **Figure 1**.

**Figure 2:**
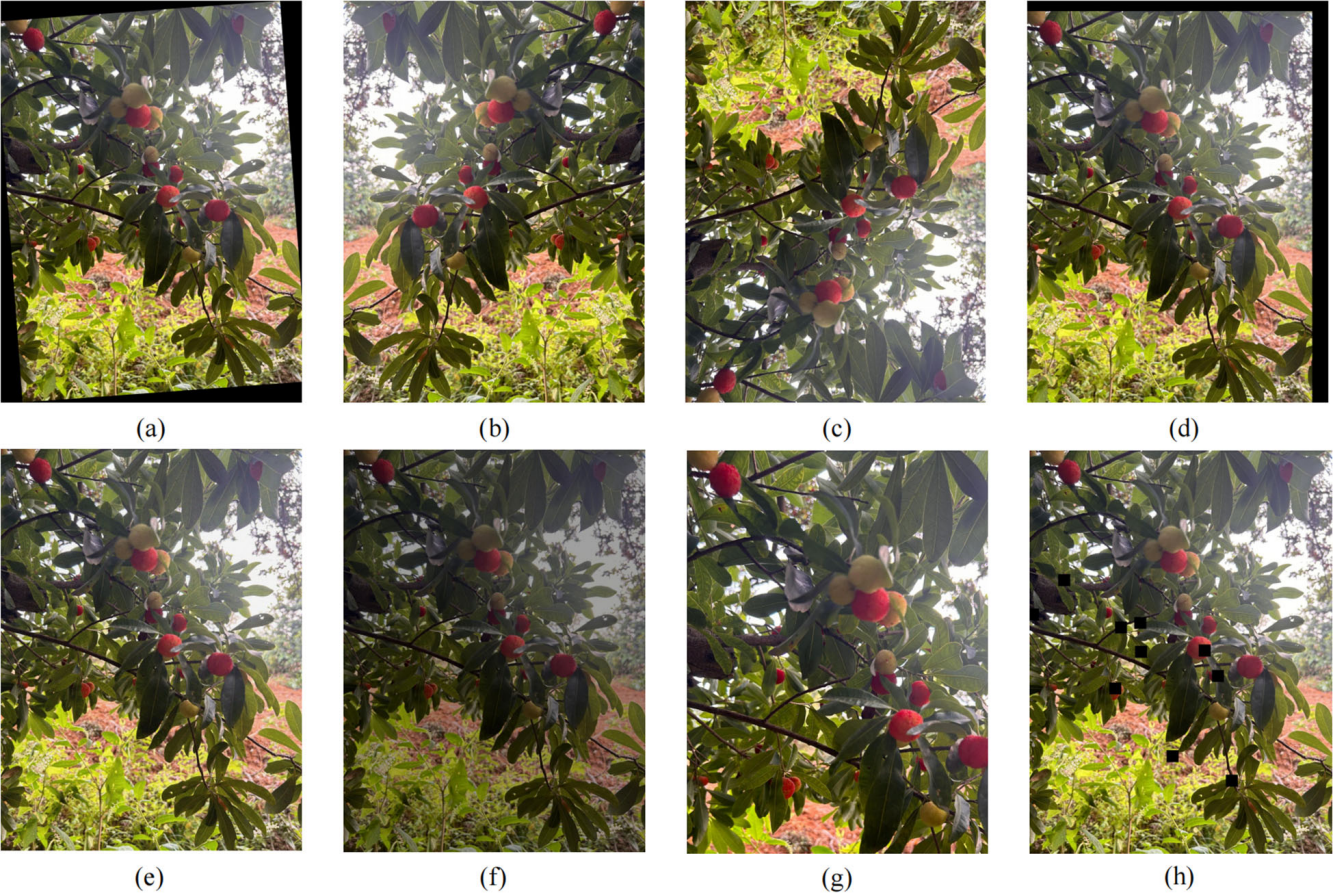
Data augmentation effects **(a)** random rotation; **(b)** horizontal flip; **(c)** vertical flip; **(d)** random translation; **(e)** Gaussian blur; **(f)** brightness adjustment; **(g)** random crop; **(h)** local occlusion enhancement.

To address the relatively limited sample size of the bayberry dataset and improve model recognition performance in complex environments while effectively alleviating overfitting, this study adopted offline data augmentation strategies to preprocess original images. From the originally collected 2,512 images, we rigorously screened and removed duplicate, blurred, and overexposed low-quality images, ultimately obtaining 1,954 high-quality images to form the base dataset. The screening process particularly emphasized retaining images with different lighting conditions, shooting angles, and background environments to ensure good quality and diversity of the dataset. The dataset was divided into training (1,367 images), validation (391 images), and test sets (196 images) in a 7:2:1 ratio, and all images were precisely annotated using the image annotation tool LabelImg, drawing completely surrounding rectangular bounding boxes for each bayberry target. To further expand the data scale, data augmentation (Shorten and Khoshgoftaar, 2019) was applied to the training set, expanding the sample size from 1,367 to 5,468 images: in terms of geometric transformations, random rotation, horizontal/vertical flipping, and random translation operations were implemented to effectively simulate multi-angle shooting scenarios; in terms of image quality enhancement, dynamic adjustment of image brightness and addition of Gaussian noise enhanced the model’s adaptability to different lighting conditions and imaging quality. The above diversified data augmentation strategies not only effectively expanded the dataset scale but more importantly increased data diversity, providing strong support for stable model performance in complex orchard environments. The augmentation effects are shown in **Figure 2**.

### 2.2 RT-DETR baseline framework

RT-DETR represents a major breakthrough in real-time object detection, successfully addressing the computational limitations of traditional DETR models while maintaining their end-to-end detection advantages. The model’s outstanding performance stems from its innovative architecture, eliminating the need for Non-Maximum Suppression (NMS) post-processing while achieving competitive speed and accuracy metrics compared to mature YOLO variants. RT-DETR’s strength lies in its ability to simultaneously process entire images through Transformer self-attention mechanisms, enabling better understanding of global context compared to traditional CNN-based methods that process images through local receptive fields.

The RT-DETR architecture consists of three main components that work synergistically to achieve optimal detection performance. The backbone network, typically based on ResNet (He et al., 2016) variants, serves as the foundation for feature extraction, processing input images through multiple stages to generate hierarchical feature representations at different scales. The neck network implements an efficient hybrid encoder that processes multi-scale features through intra-scale interaction and cross-scale fusion mechanisms, effectively combining features from different resolution levels while maintaining computational efficiency. The detection head employs a Transformer decoder with auxiliary prediction heads, utilizing IoU-aware query selection to initialize object queries and iteratively optimizing detection results through multiple decoder layers. This architecture enables flexible inference speed adjustment by changing the number of decoder layers without retraining the model, making it particularly suitable for deployment in resource-constrained agricultural environments.

### 2.3 Related research on feature enhancement and adaptive fusion techniques in small target detection

Enhancing feature extraction capabilities for small target detection has always been a core challenge in computer vision applications. Zhang et al. proposed a Multi-Resolution Attention Extractor, effectively improving small target detection performance in remote sensing images through multi-scale feature extraction and attention mechanism fusion (Zhang et al., 2020). Qiu et al. proposed YOLO-SDL, a lightweight wheat grain detection technology based on improved YOLOv8n, demonstrating effective small target detection capabilities (Qiu et al., 2024). Wang et al. developed FE-YOLOv5, a feature enhancement network that designed Feature Enhancement Module (FEM) to capture more discriminative small target features, significantly improving small target detection through global attention and high-level global contextual information guiding shallow high-resolution features (Wang et al., 2023). Zhang et al. constructed a multi-granularity deformable convolution-based Feature Enhancement Network (FENet) that effectively addressed the problem of small targets being susceptible to scale variations by learning and capturing variations in target shape and scale to obtain offset feature representations at different granularities (Zhang et al., 2023). These studies demonstrate that multi-path feature enhancement mechanisms and cross-stage partial connection techniques show significant advantages in improving gradient flow and feature representation quality.

Multi-scale feature fusion methods have evolved from simple concatenation approaches to complex attention-based mechanisms capable of selectively emphasizing relevant features at different scales. Ren et al. proposed an interactive multi-scale feature representation enhancement strategy that effectively bridged the gap between small target and medium-large target detection performance through multi-scale auxiliary enhancement networks achieving feature interaction under different inputs (Ren et al., 2017). Wei et al. developed SED-YOLO, a multi-scale attention model that maintained key information while reducing computational costs by replacing standard convolution with Switchable Atrous Convolution (SAC) and introducing Efficient Multi-scale Attention (EMA) mechanisms (Wei et al., 2025). Huang et al. designed a multi-scale feature fusion convolutional neural network that significantly improved regional feature expression capabilities for indoor small target detection through fusion of candidate region features at different levels and normalization processing (Huang et al., 2022). These works demonstrate that the integration of spatial and frequency domain information processing has become a powerful technique for enhancing feature discrimination capabilities.

## 3 Methodology

**Figure 3:**
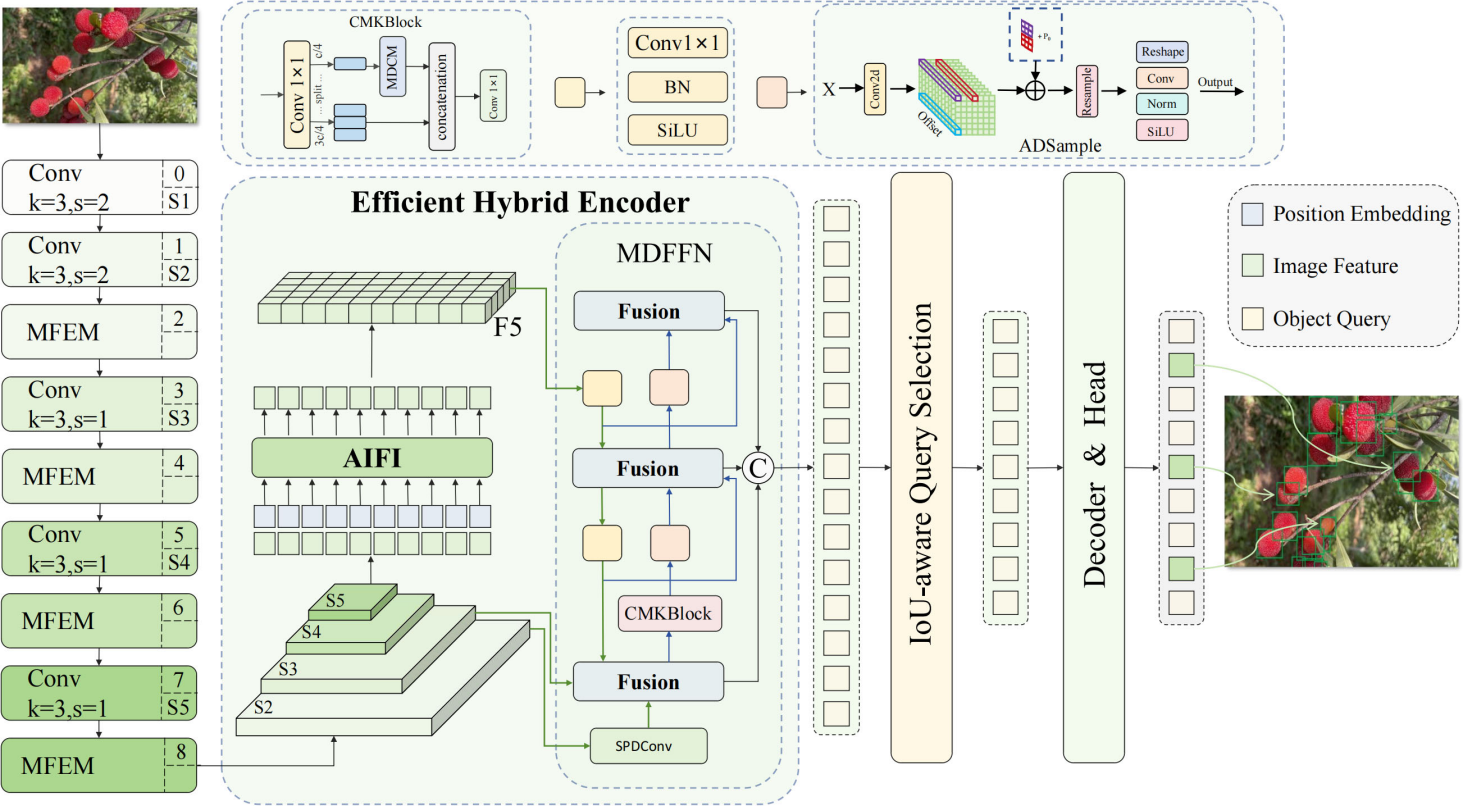
MDE-DETR model architecture.

The Multi-Domain Enhanced DETR (MDE-DETR) algorithm proposed in this paper addresses the technical challenges of small-target bayberry detection in complex orchard environments by constructing an end-to-end detection framework based on multi-domain enhanced feature fusion. The framework contains three core innovations: First, the Enhanced Feature Extraction Network (EFENet) is designed as the backbone architecture, significantly improving the network’s perception capability for small target features through Multi-Path Feature Enhancement Module (MFEM) and reparameterized convolution techniques; Second, the Multi-Domain Feature Fusion Network (MDFFN) architecture is proposed, integrating SPDConv spatial pixel rearrangement, Cross-Stage Multi-Kernel Block (CMKBlock), and Dual-Domain Attention Module (DDAM) to achieve efficient multi-scale feature fusion; Finally, the Adaptive Deformable Sampling (ADSample) downsampling module is constructed, effectively preserving key feature information during downsampling through learnable spatial offset prediction mechanisms. The overall MDE-DETR framework structure is shown in **Figure 3**, with the three innovative modules working synergistically to provide an efficient and reliable technical solution for small-target bayberry detection in complex environments.

### 3.1 Enhanced feature extraction network

Traditional ResNet backbone networks expose numerous limitations in small-target bayberry detection tasks in complex orchard environments. The single convolutional path of ResNet blocks limits the diversity of feature representation, making it difficult to simultaneously capture target features at different scales and orientations, particularly performing poorly when facing complex branch and leaf texture interference. Additionally, ResNet architecture lacks explicit modeling of inter-channel dependencies, unable to effectively distinguish foreground targets from background noise, leading to decreased detection accuracy under illumination changes and occlusion conditions. To address these challenges, we propose the EFENet backbone network based on MFEM modules, significantly enhancing the network’s perception capability for small target features and adaptability to complex environments through integration of reparameterized convolution, cross-stage partial connections, and enhanced local attention mechanisms, providing efficient and reliable technical support for precision agriculture detection.

**Figure 4:**
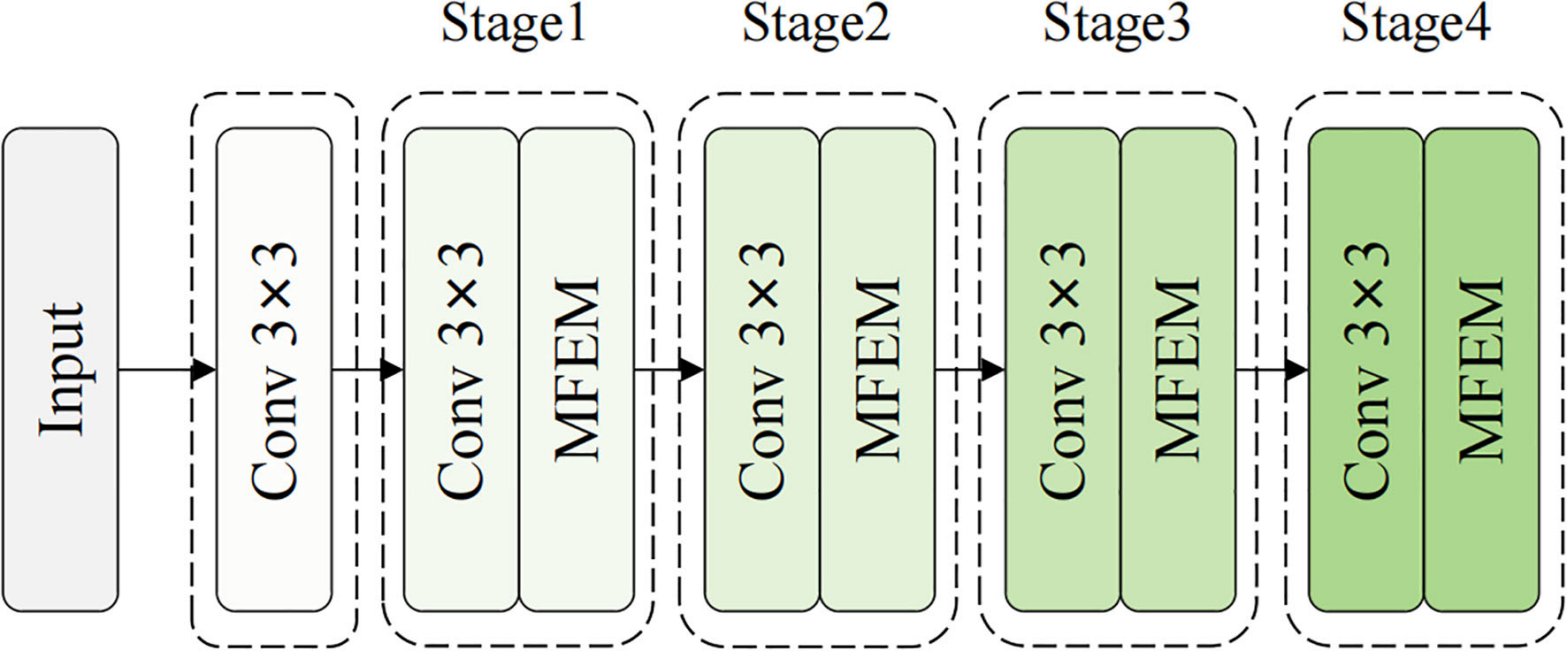
EFENet backbone network structure.

The EFENet backbone network is divided into four main stages, corresponding to feature scales of P2/4, P3/8, P4/16, and P5/32, as shown in **Figure 4**. Compared to traditional ResNet’s unidirectional residual propagation, EFENet achieves multi-path feature flow and cross-stage information interaction through MFEM modules, effectively enhancing gradient propagation efficiency and feature reuse capability. Meanwhile, the network maintains deep learning expression capabilities while achieving multi-branch structure during training and single-branch efficient execution during inference through reparameterization techniques, laying a solid foundation for mobile deployment and real-time detection applications.

**Figure 5:**
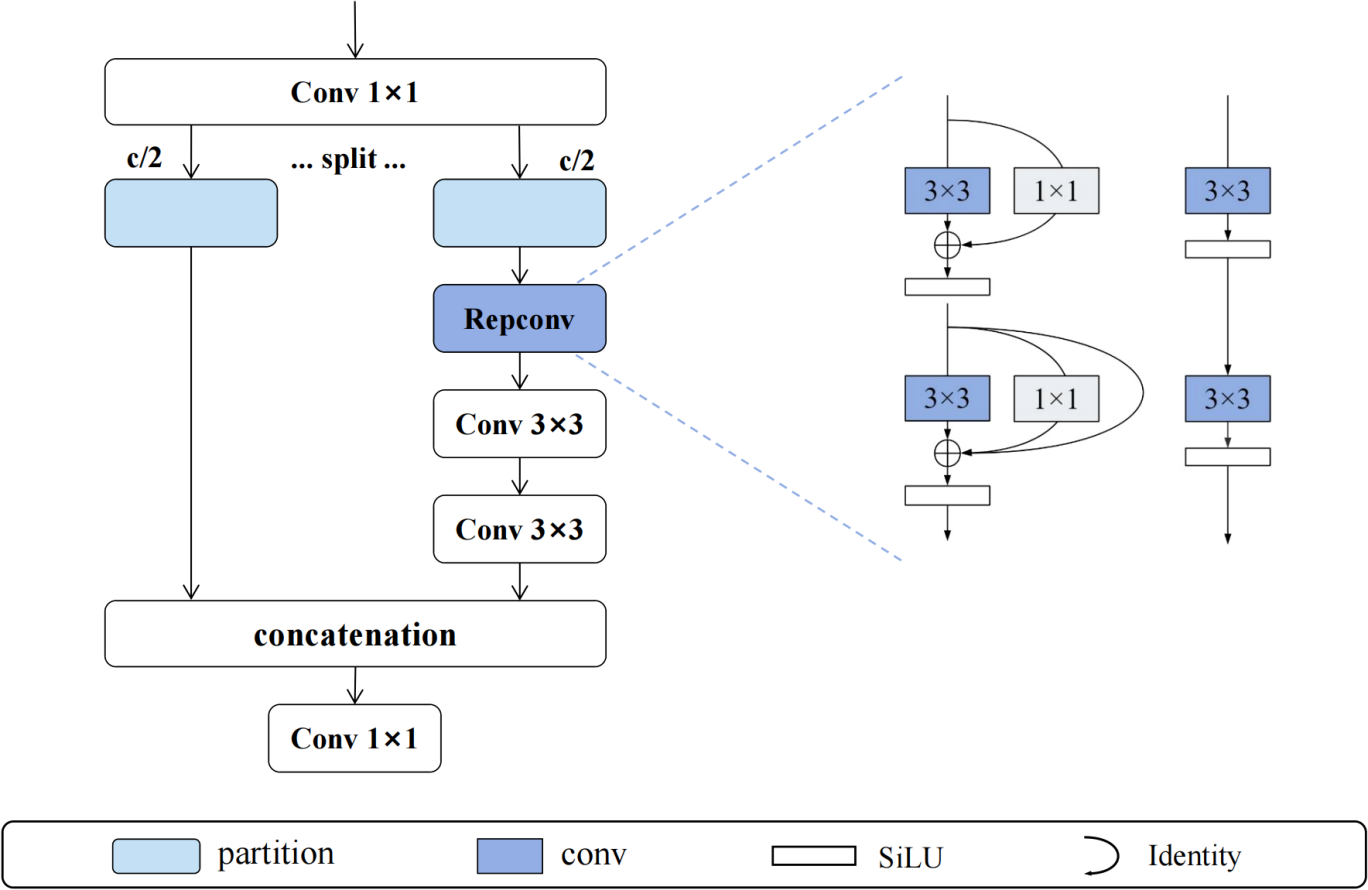
MFEM module structure.

The MFEM module is based on the design concept of cross-stage partial connections, achieving efficient gradient-aware feature learning through three stages: feature splitting, parallel processing, and adaptive fusion. As shown in **Figure 5**, the module first uses 1×1 convolution to expand input features along the channel dimension, which are then equally divided into two independent processing branches F_1_ and F_2_. The F_1_ branch maintains the integrity of original feature information, while the F_2_ branch undergoes deep feature extraction through RepConv reparameterized convolution, generating F_rep. Subsequently, F_rep is processed through n-1 consecutive 3×3 convolution layers in cascade, with each convolution layer learning different levels of abstract feature representations. Finally, the module adjusts the feature dimensions of the last layer through 1×1 convolution and performs adaptive fusion of all branch outputs. The output of the entire MFEM module can be expressed as shown in Equation 1:

(1) $$Y=W_{cv2}\;*\;\text{Concat}[F_{1},F_{rep},F_{m1},\ldots ,F_{mn-1},F_{cv4}]$$

where ${\text{F}}_{\text{mi}}$ represents the output feature of the i-th cascaded convolution, ${\text{F}}_{\text{cv}4}={\text{W}}_{\text{cv}4}*{\text{F}}_{\text{mn}-1}+{\text{b}}_{\text{cv}4}$ is the adjustment result of the final 1×1 convolution, and Concat[·]represents feature concatenation along the channel dimension. This multi-branch parallel processing and hierarchical feature fusion mechanism enables MFEM to significantly enhance the richness and discriminative capability of feature expression while maintaining computational efficiency.

RepConv (Reparameterized Convolution) (Ding et al., 2021) modules employ structural reparameterization techniques to achieve perfect unity between multi-branch learning during training and single-branch execution during inference. During the training phase, RepConv performs collaborative feature learning through three parallel branches: the main branch uses 3×3 convolution kernels to capture local spatial feature relationships, the auxiliary branch uses 1×1 convolution kernels for cross-channel information interaction, and the identity mapping branch (implemented through BatchNorm) maintains the transmission of original feature information. The forward propagation process during training can be mathematically expressed as shown in Equation 2:

(2) $$F_{train}=\sigma (W_{3\times 3}*X+b_{3\times 3}+W_{1\times 1}*X+b_{1\times 1}+I_{bn})$$

where $W_{3\times 3}\text{ and }W_{1\times 1}$represent 3×3 and 1×1 convolution kernel parameters respectively, $b_{3\times 3}\text{ and }b_{1\times 1}$are corresponding bias terms, *I_bn_* represents the output of the BatchNorm branch, *σ* is the SiLU activation function, and *represents convolution operation.

During inference, RepConv reparameterizes the multi-branch structure into a single 3×3 convolution operation through mathematically equivalent transformations, involving complex parameter fusion calculations. The core of reparameterization lies in unifying convolution kernels of different sizes to the same spatial dimensions and integrating BatchNorm parameters into convolution weights and biases. Specifically, 1×1 convolution kernels are extended to 3×3 size through zero padding, and identity mapping is represented as equivalent 3×3 convolution through diagonal unit matrix construction. The final equivalent convolution parameters are calculated through Equations 3 and 4:

(3) $$W_{equiv}=W_{3\times 3}^{fused}+{\text{Pad}}_{3\times 3}(W_{1\times 1}^{fused})+W_{identity}^{fused}$$

(4) $$b_{equiv}=b_{3\times 3}^{fused}+b_{1\times 1}^{fused}+b_{identity}^{fused}$$

where the superscript *fused* represents convolution weights and biases that have integrated BatchNorm parameters, ${\text{Pad}}_{3\times 3}(\cdot )$represents the operation of extending 1×1 convolution kernels to 3×3 size through zero padding,and $W_{identity}^{fused},b_{identity}^{fused}$ are the equivalent convolution parameters of the identity mapping branch. Through this reparameterization mechanism, RepConv maintains rich feature learning capabilities during training while achieving efficient single-branch execution during inference, providing ideal performance guarantees for practical deployment applications.

The EFENet backbone network achieves significant performance breakthroughs in small-target bayberry detection tasks in complex orchard environments through the synergistic effects of MFEM modules and RepConv techniques. This network architecture effectively overcomes the limitations of traditional ResNet in feature expression diversity and computational efficiency, significantly enhancing the network’s perception capability for fine-grained target features and robustness against complex environmental interference through deep integration of cross-stage partial connections and reparameterized convolution.

### 3.2 Multi-domain feature fusion network

**Figure 6:**
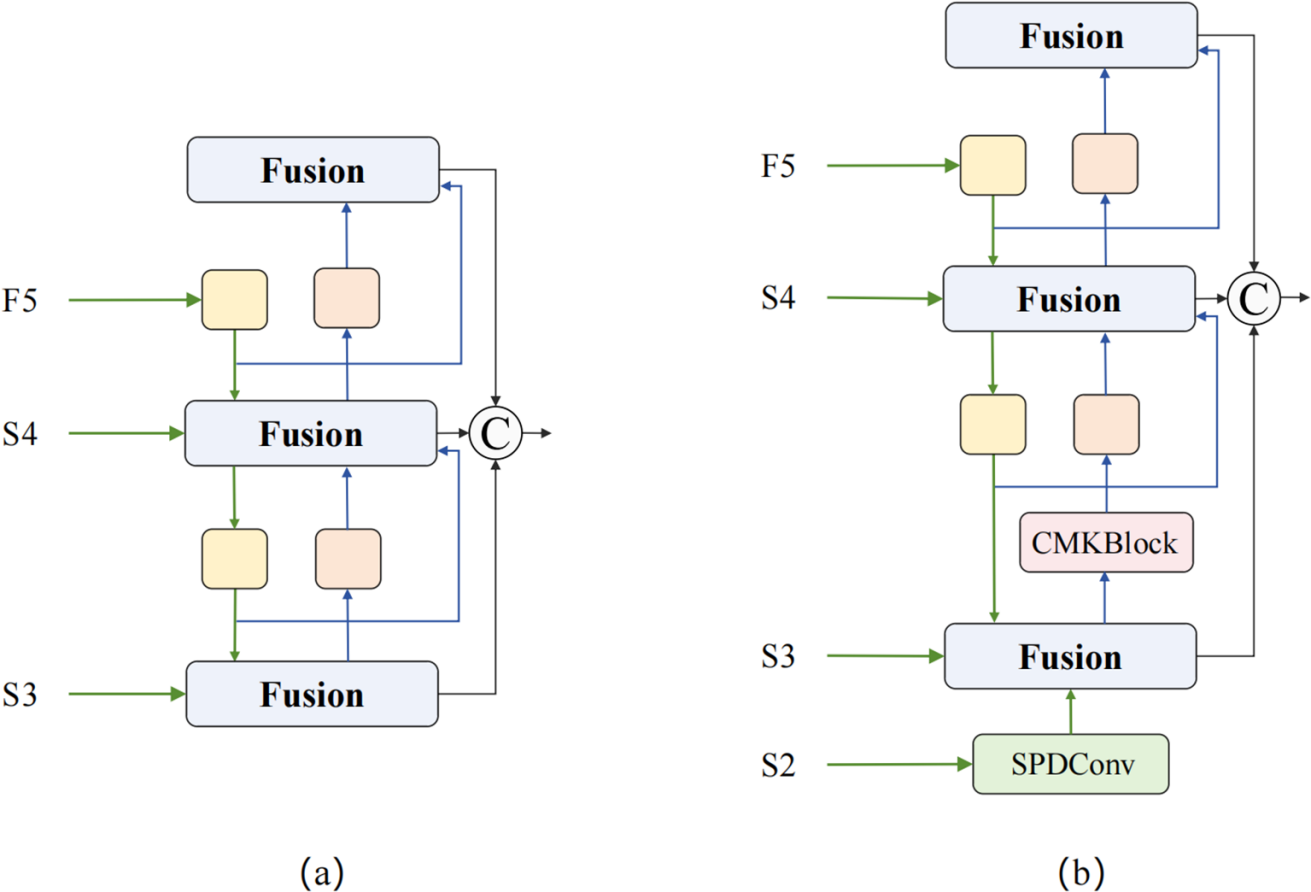
CCFM and MDFFN structure comparison. **(a)** CCFM; **(b)** MDFFN.

Traditional Concatenation-Convolution Feature Fusion (CCFM) architecture adopts linear fusion methods, lacking effective utilization of frequency domain information and unable to fully exploit nonlinear correlations between features. It inevitably causes information loss during downsampling processes, particularly causing irreversible damage to detail features of small targets. To address these problems, we propose the MDFFN feature fusion architecture (as shown in **Figure 6**), which significantly improves the effectiveness of multi-scale feature fusion and the accuracy of small target detection while maintaining optimal computational efficiency balance through integration of spatial-channel lossless transformation, omnidirectional kernel feature enhancement, and dual-domain feature propagation mechanisms.

The MDFFN feature fusion architecture first employs SPDConv (Sunkara and Luo, 2023) spatial pixel rearrangement modules to achieve lossless conversion between spatial resolution and channel dimensions through regular pixel reorganization strategies while reducing computational complexity and completely preserving detail information. Subsequently, CMKBlock omnidirectional kernel feature enhancement modules are introduced, achieving adaptive fusion of features with different receptive fields and significant improvement in expression capabilities through the synergistic effects of cross-stage partial connection strategies and multi-scale parallel convolution kernels. Finally, DDAM and Frequency Modulation Block (FMB) are integrated to perform feature optimization simultaneously in spatial and frequency domains, fully utilizing the global receptive field characteristics of Fourier transform and rich information representation capabilities of complex domains to achieve more precise feature modulation and stronger environmental adaptability.

**Figure 7:**
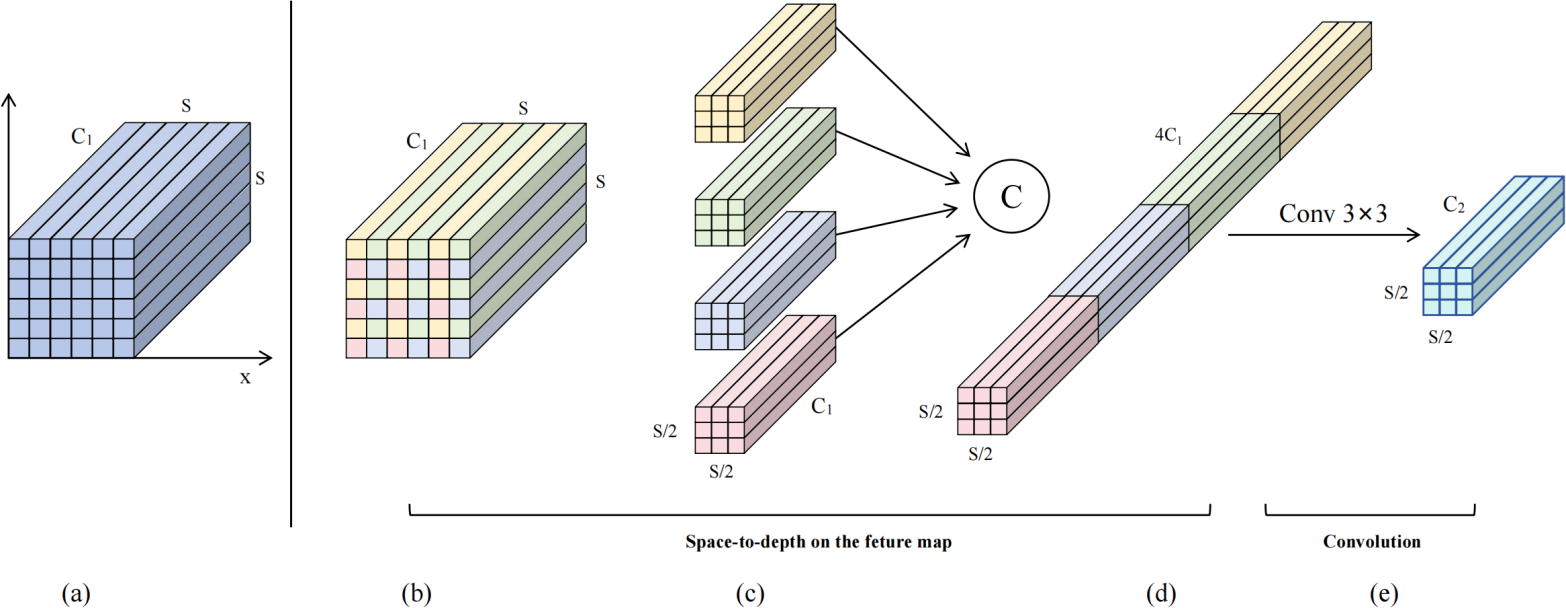
SPDConv schematic diagram when scale=2. **(a)** Input feature map; **(b-d)** Space-to-depth conversion; **(e)** Output feature map.

The Space-to-Depth Convolution (SPDConv) module employs spatial pixel rearrangement strategies to achieve efficient conversion between spatial resolution and channel dimensions of feature maps through lossless dimensional transformation. The core idea of this module is to re-encode spatial information of input feature maps into channel dimensions, avoiding information loss caused by traditional pooling or stride convolution. The specific workflow (as shown in **Figure 7**): For input feature map X∈ℝ^(C×H×W), pixel rearrangement is performed according to a 2×2 grid pattern, decomposing the feature map into four independent sub-feature maps corresponding to pixel sampling results at top-left (0,0), top-right (0,1), bottom-left (1,0), and bottom-right (1,1) positions. These four sub-feature maps are concatenated along the channel dimension, expanding the output feature channels to 4 times the original while halving the spatial resolution. Subsequently, the concatenated high-dimensional features undergo deep processing and channel number adjustment through 3×3 convolution, finally outputting feature representations of target dimensions. The entire mathematical transformation process of SPDConv can be expressed as shown in Equation 5:

(5) $$F_{SPD}=W_{3\times 3}*\text{Concat}[X_{\left[::2,::2\right]},X_{\left[1::2,::2\right]},X_{\left[::2,1::2\right]},X_{\left[1::2,1::2\right]}]+b$$

where $X_{\left[i::2,j::2\right]}$ represents sub-feature maps sampled every 2 pixels starting from position (i,j),and $W_{3\times 3}\in {\text{R}}^{C_{out}\times 4C\times 3\times 3}$and b are learnable convolution weights and bias parameters respectively.

**Figure 8:**
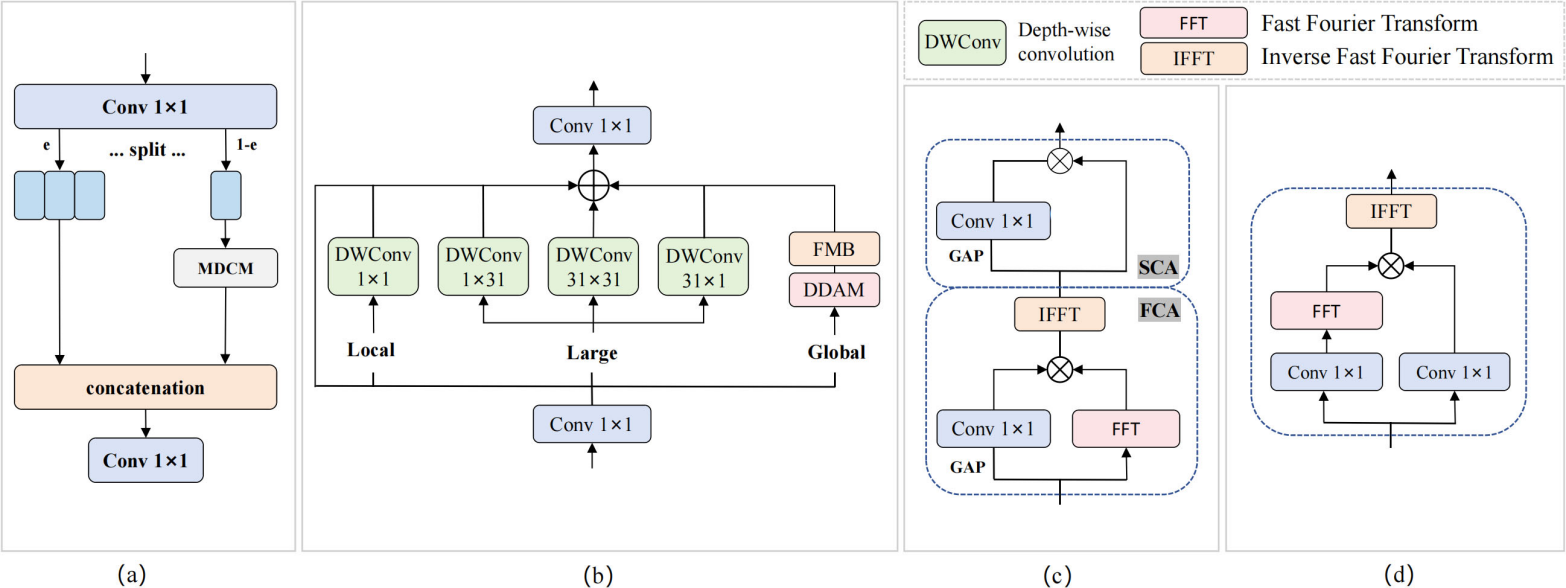
CMKBlock module structure. **(a)** CMKBlock; **(b)** MDCM; **(c)** DDAM; **(d)** FMB.

The CMKBlock module is based on the design concept of cross-stage partial connections, achieving efficient feature learning through feature branch processing and omnidirectional kernel enhancement, as shown in **Figure 8**. The module first preprocesses input features through 1×1 convolution, then splits the processed features into two functionally different branches. The smaller branch (25% channels) serves as the processing branch, undergoing deep feature enhancement through the embedded Multi-Directional Convolution Module (MDCM); the larger branch (75% channels) serves as the identity branch, directly maintaining the transmission of original feature information, thereby reducing computational complexity and enhancing gradient flow effects. Finally, adaptive fusion of the two branches is achieved through feature concatenation and 1×1 convolution.

The MDCM module achieves omnidirectional feature enhancement through multi-scale parallel convolution and dual-domain attention mechanisms. This module integrates four different geometric shapes of depth convolution kernels (1×1 point convolution, 1×31 horizontal strip convolution, 31×1 vertical strip convolution, 31×31 standard convolution) to capture feature patterns in different directions and scales. Dual-domain attention mechanisms and frequency domain gating modules are simultaneously introduced for feature optimization. The output features of MDCM are obtained through multi-path residual connections and nonlinear activation, as shown in Equation 6:

(6) $$F_{MDCM}=\sigma (X+\sum_{k\in \{1\times 1,1\times 31,31\times 1,31\times 31\}}W_{k}*F_{in}+F_{DDAM})$$

where ${\text{F}}_{\text{in}}=\text{GELU}({\text{W}}_{\text{in}}*\text{X})$ is the input preprocessing result, ${\text{W}}_{\text{k}}$represents depth convolution weights of corresponding geometric shapes, ${\text{F}}_{\text{DDAM}}$is the output of the dual-domain attention mechanism, and σis the ReLU activation function.

DDAM achieves precise feature modulation through cascaded processing of Frequency Channel Attention (FCA) and Spatial Channel Attention (SCA). FCA first generates frequency domain attention weights using global average pooling and convolution, then performs feature weighting in the frequency domain; SCA performs further spatial domain attention modulation on FCA outputs. The entire DDAM processing flow embodies synergistic optimization of frequency and spatial domains, as described in Equation 7:

(7) $$F_{DDAM}=W_{sca}*\text{GAP}(F_{fca})⊙F_{fca}$$

where $F_{fca}=|{\text{F}}^{-1}(W_{fca}*\text{GAP}(F_{in})⊙\text{F}(F_{in}))|$, F(·) and ${\text{F}}^{-1}(\cdot )$represent two-dimensional fast Fourier transform and inverse transform respectively, GAP(·) represents global average pooling operation, and ⊙ represents element-wise multiplication.

FMB achieves adaptive feature enhancement through nonlinear modulation in complex domains, containing two parallel 1×1 convolution branches and two learnable channel-level modulation parameters α and β. The specific workflow: input features obtain F_1_ and F_2_ through two convolution branches respectively, F_2_ undergoes Fourier transform and then performs element-wise multiplication with F_1_ in the complex domain, finally recovering to spatial domain through inverse Fourier transform combined with residual connections. The mathematical expression of the entire FMB is shown in Equation 8:

(8) $$F_{FMB}=\alpha ⊙|F^{-1}(W_{1}*X⊙F(W_{2}*X))|+\beta ⊙X$$

where ⊙ represents element-wise multiplication, $\text{α},\text{β}\in {\text{R}}^{\text{C}\times 1\times 1}$are learnable channel-level modulation parameters, and W_1_, W_2_ are weight parameters of two 1×1 convolutions.

The MDFFN feature fusion architecture achieves significant performance breakthroughs in small-target bayberry detection tasks in complex orchard environments through deep integration of SPDConv, CMKBlock, and dual-domain attention mechanisms. This architecture successfully overcomes the inherent limitations of traditional CCFM feature fusion methods in information preservation, multi-scale processing, and computational efficiency, significantly improving the network’s capability to preserve detail information and perceive targets of different geometric shapes through lossless spatial-channel dimension conversion and omnidirectional kernel feature enhancement. The SPDConv module’s pixel rearrangement strategy completely eliminates information loss problems in traditional downsampling, while the CMKBlock module’s multi-kernel parallel processing and dual-domain attention mechanisms achieve more precise feature representation and stronger background interference suppression capabilities.

### 3.3 Adaptive deformable sampling

**Figure 9:**
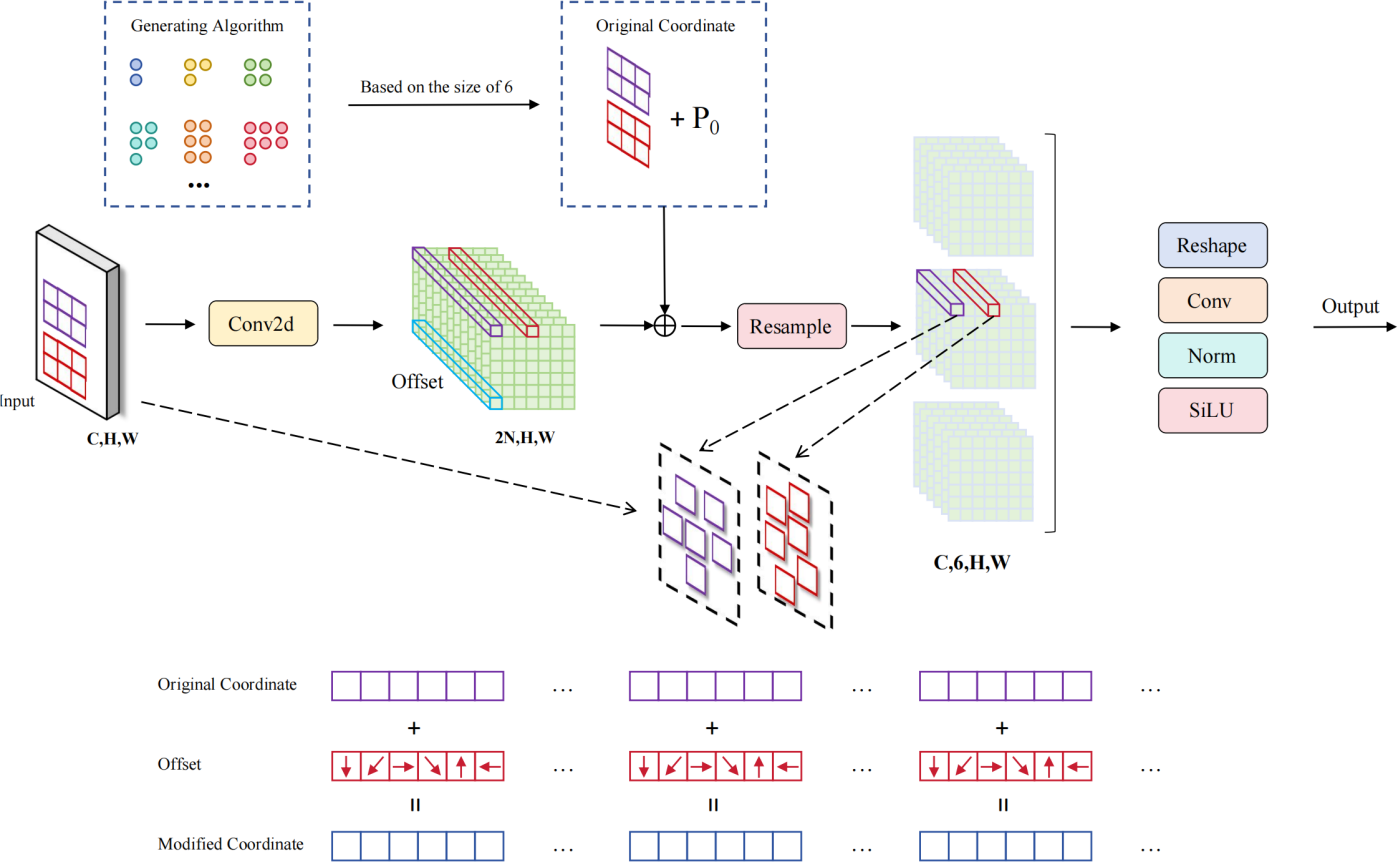
ADSample module structure.

Traditional 3×3 stride convolution downsampling methods have significant limitations in small-target bayberry detection in complex orchard environments. First, fixed convolution kernel sampling patterns cannot adaptively adjust sampling positions, leading to loss of important target feature information during downsampling processes. Second, traditional downsampling methods lack perception capabilities for target geometric shapes, unable to flexibly adjust receptive fields to adapt to different spatial layout patterns when facing complex fruit distributions and occlusion situations. To address these problems, we propose the ADSample learnable deformable downsampling module (as shown in **Figure 9**), which enables downsampling processes to dynamically adjust sampling positions according to spatial distributions of input features through introduction of adaptive offset learning mechanisms and bilinear interpolation resampling strategies, effectively preserving key feature information of small targets and significantly improving the network’s perception capability for irregular targets in complex orchard environments and precision of feature expression.

The ADSample module achieves intelligent downsampling operations through learnable spatial offset prediction and adaptive feature resampling, with the core design concept of enabling networks to autonomously learn optimal sampling strategies to adapt to different target feature distributions. The module first uses an offset prediction network composed of a 3×3 convolution layer to process input features X∈ℝ^(C×H×W), generating spatial offsets with 2N channels, where N represents the number of sampling points corresponding to each output position. The offset prediction process predicts optimal sampling position offsets by learning local spatial patterns of input features, which can be mathematically expressed as shown in Equation 9:

(9) $$\Delta =W_{offset}*X+b_{offset}$$

where ${\text{W}}_{\text{offset}}\in {\text{R}}^{2\text{N}\times \text{C}\times 3\times 3}$ are convolution weight parameters of the offset prediction network, ${\text{b}}_{\text{offset}}$ are corresponding bias parameters,and $\text{Δ}\in {\text{R}}^{2\text{N}\times \text{H}\times \text{W}}$ is the predicted two-dimensional spatial offset tensor.

Next, the module constructs final sampling coordinates by combining regular sampling grids, predefined sampling patterns, and learned offsets. The baseline sampling grid P_0_ is uniformly distributed on input feature maps according to downsampling strides, and the predefined sampling pattern P_n_ defines initial sampling point layouts relative to baseline positions. By adding these components, final coordinates for each sampling point are obtained, reflecting the transition from regular sampling to adaptive sampling, as shown in Equation 10:

(10) $$P=P_{0}+P_{n}+\Delta$$

where ${\text{P}}_{0}\in {\text{R}}^{2\text{N}\times \text{H}\times \text{W}}$ represents regular sampling baseline grids corresponding to output strides, ${\text{P}}_{\text{n}}\in {\text{R}}^{2\text{N}\times 1\times 1}$ is relative sampling position templates generated based on $\sqrt{\text{N}}\times \sqrt{\text{N}}$ grid patterns, $\text{P}\in {\text{R}}^{2\text{N}\times \text{H}\times \text{W}}$ are final adaptive sampling coordinates.

Since predicted sampling positions are typically non-integer coordinates, the module employs bilinear interpolation methods for feature sampling in continuous space. For any sampling point $\text{p}=({\text{p}}_{\text{x}},{\text{p}}_{\text{y}})$, the algorithm first determines four integer coordinate neighbors around it, then calculates corresponding interpolation weights. Taking the top-left neighbor as an example, its interpolation weight calculation considers distance relationships between sampling points and neighbor points in both spatial dimensions, as shown in Equation 11:

(11) $$w_{lt}=\max(0,1-|p_{x}-\lfloor p_{x}\mathrm{=“\rfloor}|)\times \max(0,1-|p_{y}-\lfloor p_{y}\mathrm{=“\rfloor}|)$$

Similarly, interpolation weights for bottom-right ${\text{w}}_{\text{rb}}$, bottom-left ${\text{w}}_{\text{lb}}$, and top-right ${\text{w}}_{\text{rt}}$ can be calculated, ensuring continuity and differentiability of the sampling process.

The feature resampling process is achieved through bilinear interpolation, where feature values at each sampling position are obtained by weighted summation of feature values from four integer neighbors. This process not only ensures sampling smoothness but also maintains effective gradient propagation, enabling end-to-end training of the entire module, as described in Equation 12:

(12) $$F_{resampled}(p)=\sum_{\left(i,j)\in \{lt,rb,lb,rt\right\}}w_{ij}\cdot X(q_{ij})$$

where ${\text{q}}_{\text{ij}}$ represents four integer coordinate neighbors around sampling point p, and ${\text{w}}_{\text{ij}}$ are corresponding bilinear interpolation weights.

Finally, resampled features ${\text{F}}_{\text{resampled}}\in {\text{R}}^{\text{C}\times \text{H}\times \text{W}\times \text{N}}$ undergo special spatial rearrangement operations, stacking information from N sampling points along height dimensions to form tensor formats suitable for column convolution processing. Subsequently, feature integration and dimensional transformation are achieved through a convolution layer with kernel size (N;1), realizing final downsampling output, as shown in Equation 13:

(13) $$Y=\sigma (W_{col}*\text{Rearrange}(F_{resampled})+b_{col})$$

where ${\text{W}}_{\text{col}}\in {\text{R}}^{{\text{C}}_{\text{out}}\times \text{C}\times \text{N}\times 1}$ are weight parameters of column convolution, Rearrange(·) represents operations that rearrange resampled features from (C,H,W,N) to (C,H×N,W) format, and σ is the SiLU activation function.

The ADSample learnable deformable downsampling module achieves significant performance improvements in small-target bayberry detection tasks in complex orchard environments through synergistic effects of adaptive offset learning and bilinear interpolation resampling. This module successfully overcomes limitations of traditional fixed convolution downsampling in feature preservation and geometric adaptability, enabling downsampling processes to better adapt to geometric features and spatial distributions of targets through dynamic adjustment of sampling positions.

## 4 Experimental results and analysis

### 4.1 Experimental setup

ll experiments in this study were conducted on a high-performance computing platform to ensure reliability and reproducibility of experimental results. The detailed experimental configuration is shown in **Table 1**. The hardware environment includes an Intel Core i9-10900K processor, 32GB memory, and an NVIDIA GeForce RTX 3090 graphics card, providing sufficient computational resources for deep learning model training and inference. The software environment is based on Python 3.9 and PyTorch deep learning framework. During the training process, the batch size was set to 8, training epochs to 300, AdamW optimizer was used, and the initial learning rate was set to 0.0001.

**Table 1:** Experimental configuration.

Configuration item	Parameter
Operating System	Windows 10
Processor	Intel(R) Core(TM) i9-10900K@ 3.70GHz
Memory	32 GB
Graphics Card	NVIDIA GeForce RTX 3090
Programming Language	Python 3.9
Deep Learning Framework	PyTorch
Training Framework	8
Epochs	300
Optimizer	AdamW
Initial Learning Rate	0.0001

### 4.2 Evaluation metrics

To comprehensively evaluate model performance and efficiency in bayberry detection tasks, Precision (P), Recall (R), and mean Average Precision (mAP) are used to measure model performance. Precision reflects the accuracy of model prediction results, i.e., the proportion of true positive samples among all results predicted as positive samples.

Recall reflects the completeness of model prediction results, i.e., the proportion of correctly predicted positive samples among all actual positive samples. mAP is the area under the precision-recall curve at different Intersection over Union thresholds, serving as a comprehensive performance indicator considering both precision and recall. The calculation methods for evaluation metrics are shown in Equations 14–17.

(14) $$P=\frac{T_{P}}{F_{N}+F_{P}}\times 100\%$$

(15) $$R=\frac{T_{P}}{T_{P}+F_{N}}\times 100\%$$

(16) $$\text{AP}=\int_{0}^{1}P(R)dR$$

(17) $$\text{mAP}=\frac{\sum_{i=1}^{N}{\text{AP}}_{i}}{N}\times 100\%$$

where *T_P_* is the number of positive samples predicted as positive; *F_P_* is the number of negative samples predicted as positive; *F_N_* is the number of positive samples predicted as negative.

In terms of model complexity, model parameters, Floating Point Operations (FLOPs), and memory usage are used as measurement indicators. Model parameters reflect the scale and complexity of the model. FLOPs reflect the computational load of the model during inference. Memory usage reflects the model’s demand for memory resources during operation.

### 4.3 Experimental results analysis

#### 4.3.1 Comparison experiments of different backbones

To verify the effectiveness of the proposed EFENet backbone network, this study compared multiple mainstream backbone networks, with comparison results shown in **Table 2**.

**Table 2:** Comparison results of different backbones.

Module	P(%)	R(%)	mAP50(%)	mAP50:95(%)	GFLOPs(%)	Params(%)
BasicNet	87.2	82.0	89.1	62.8	56.9	19.8
StripNet (Yuan et al., 2025)	87.0	81.9	89.5	64.3	47.6	14.4
ElgcaNet (Noman et al., 2024)	87.7	83.2	89.6	64.7	46.8	13.9
ApNet (Yang et al., 2025)	86.8	82.7	88.9	62.7	46.9	13.9
DtabNet (Li et al., 2025)	87.7	83.3	90.1	65.1	57.3	18.8
FcaNet (Dai et al., 2024)	86.3	80.3	88.4	61.8	52.5	15.5
EFENet(ours)	88.1	83.4	90.5	65.2	44.4	13.8

Based on RT-DETR-r18, we respectively improved the backbone network with current mainstream modules while keeping other settings unchanged. From the table data, although the introduction of ApNet and FcaNet led to decreases in mAP50, StripNet, ElgcaNet, DtabNet, and our proposed EFENet all achieved improvements in mAP50 values. Among them, our proposed EFENet backbone network achieved the highest mAP50 value, fully demonstrating the effectiveness of the proposed network structure.

#### 4.3.2 Ablation experiments

To verify the optimization effects of each module, we conducted ablation experiments on the three improvement strategies proposed in this paper using our self-constructed bayberry dataset. Results are shown in **Table 3**.

**Table 3:** Ablation experiment results.

Methods	EFENet	MDFFN	ADSample	mAP50(%)	mAP50:95(%)	GFLOP(G)	Params(M)	Size(MB)
1.base	×	×	×	89.1	62.8	56.9	19.8	38.6
2	✔	×	×	90.5	65.2	44.4	13.8	27.2
3	×	✔	×	90.5	65.1	61.2	20.4	39.8
4	×	×	✔	91.4	66.6	57.9	19.5	38.0
5	✔	✔	×	92.0	66.7	56.2	15.1	29.6
6	✔	×	✔	92.4	67.3	53.8	13.5	26.6
7	×	✔	✔	91.9	66.5	59.7	19.7	38.3
8.ours	✔	✔	✔	92.9	67.9	55.5	14.7	28.9

Experimental results show that replacing the backbone network with EFENet on the original RT-DETR model improved mAP50 and mAP50:95 by 1.4% and 2.4% respectively compared to the original model, while reducing parameters, computational load, and memory usage by 30.3%, 22%, and 29.5% respectively. Using EFENet to replace traditional ResNet18 backbone not only enhanced model feature extraction capabilities but also reduced model parameters and computational complexity. After introducing the improved MDFFN module into the RT-DETR network structure, with only increases of 0.6M parameters, 4.3G computational load, and 1.2MB memory usage, the model’s mAP50 and mAP50:95 improved by 1.4% and 2.3% respectively compared to the original model. This is because processing of P2 layer and multi-scale feature fusion were added, enhancing recognition capability for small-target bayberries, though the high resolution of P2 layer caused slight increases in parameters and computational load. After constructing ADSample alone, the model’s mAP50 and mAP50:95 improved by 2.3% and 3.8% respectively compared to the original model, with parameters, computational load, and memory usage remaining basically consistent with the original model, due to its flexible sampling position adjustment mechanism improving recognition performance in dense occlusion scenarios. In the combined MDFFN and ADSample model, mAP50 and mAP50:95 improved by 2.8 and 3.7 percentage points respectively compared to the original model, with computational load increasing by 2.8G while parameters and memory usage remaining basically unchanged. Compared to the baseline network, after improvements, mAP50 and mAP50:95 improved by 3.8% and 5.1% respectively, while parameters and memory usage decreased by 25.7% and 25.1% respectively, and computational load decreased by 1.4G. The above data fully demonstrate the effectiveness of our innovations in object detection tasks, further validating their potential in practical applications.

#### 4.3.3 Comparison experiments with different models

To comprehensively evaluate the improved MDE-DETR model, this study systematically compared it with current mainstream object detection models on the same dataset. Experimental results are presented in **Table 4**, where the MDE-DETR model demonstrates significant advantages across multiple key indicators including detection accuracy, model complexity, and practical runtime efficiency. In terms of detection accuracy, the MDE-DETR model achieved the best performance in precision, recall, and mean average precision, reaching 90.5%, 85.8%, and 92.9% mAP50 respectively. Regarding model complexity, MDE-DETR has the smallest parameter count at only 14.7M and the lowest computational cost at 55.5 GFLOPs, demonstrating excellent balance between accuracy and model lightweighting. In terms of practical runtime efficiency, MDE-DETR achieves 80.1 FPS on NVIDIA RTX 3090 GPU with 640×640 input resolution, ensuring real-time performance for agricultural deployment scenarios while maintaining the highest detection accuracy. Compared to the latest YOLO12m and DEIM-D-Fine-M, MDE-DETR achieved significant improvements of 4.8% and 4.5% in mAP50 respectively, while reducing parameters by 25.01% and 23.44% respectively and maintaining competitive inference speed (80.1 FPS vs. 129.8 FPS and 68.8 FPS). Notably, while YOLO12m achieves higher FPS (129.8), MDE-DETR demonstrates superior accuracy-efficiency trade-off with 4.8% higher mAP50 at 61.7% of its speed. Compared to the RT-DETR-r18 baseline, MDE-DETR improves mAP50 by 3.8% while achieving 25.76% fewer parameters. These results, as detailed in **Table 4**, fully validate the effectiveness of MDE-DETR’s structural optimization and design choices in achieving superior accuracy-efficiency-speed trade-offs.

**Table 4:** Comparison results of different models on bayberry dataset.

Model	BackBone	Type	P(%)	R(%)	mAP50(%)	mAP50:95(%)	GFLOP(G)	Params(M)	FPS
YOLOv5m	—	One-Stage	87.5	82.4	87.2	62.9	64.0	25.0	126.6
YOLOv8m	—	One-Stage	88.0	82.6	87.6	63.2	78.7	25.8	121.3
YOLOv9m	—	One-Stage	87.4	81.3	88.0	62.6	77.0	20.1	104.3
YOLOv10m	—	One-Stage	87.2	82.7	88.4	62.1	58.9	15.3	106.1
YOLO11m	—	One-Stage	87.2	81.5	89.2	63.5	67.6	20.2	116.4
YOLO12m	—	One-Stage	88.5	81.9	88.1	62.6	59.5	19.6	129.8
SSD	VGG16	One-Stage	83.8	73.5	82.7	50.5	271.0	23.7	21.5
Faster R-CNN	ResNet50	Two-Stage	87.5	78.2	86.8	62.3	169.0	41.3	50.2
DINO	ResNet50	DETR	89.4	83.9	91.5	63.2	223.0	47.5	27.3
DEIM-D-Fine-M	HGNetv2	DETR	86.8	81.3	88.4	62.6	56.4	19.2	68.8
D-Fine-M	HGNetv2	DETR	86.6	81.0	87.1	62.3	56.4	19.2	69.2
RT-DETR-L	HGNetv2	DETR	88.5	84.5	90.4	64.4	103.4	31.9	59.2
RT-DETR-r50	ResNet50	DETR	89.7	85.0	91.4	66.0	129.5	41.9	57.2
RT-DETR-r34	ResNet34	DETR	89.3	83.7	91.0	65.5	88.8	31.1	61.3
RT-DETR-r18	ResNet18	DETR	87.2	82.0	89.1	62.8	56.9	19.8	77.6
MDE-DETR	EFENet	DETR	90.5	85.8	92.9	67.9	55.5	14.7	80.1

#### 4.3.4 Model detection effect visualization

**Figure 10:**
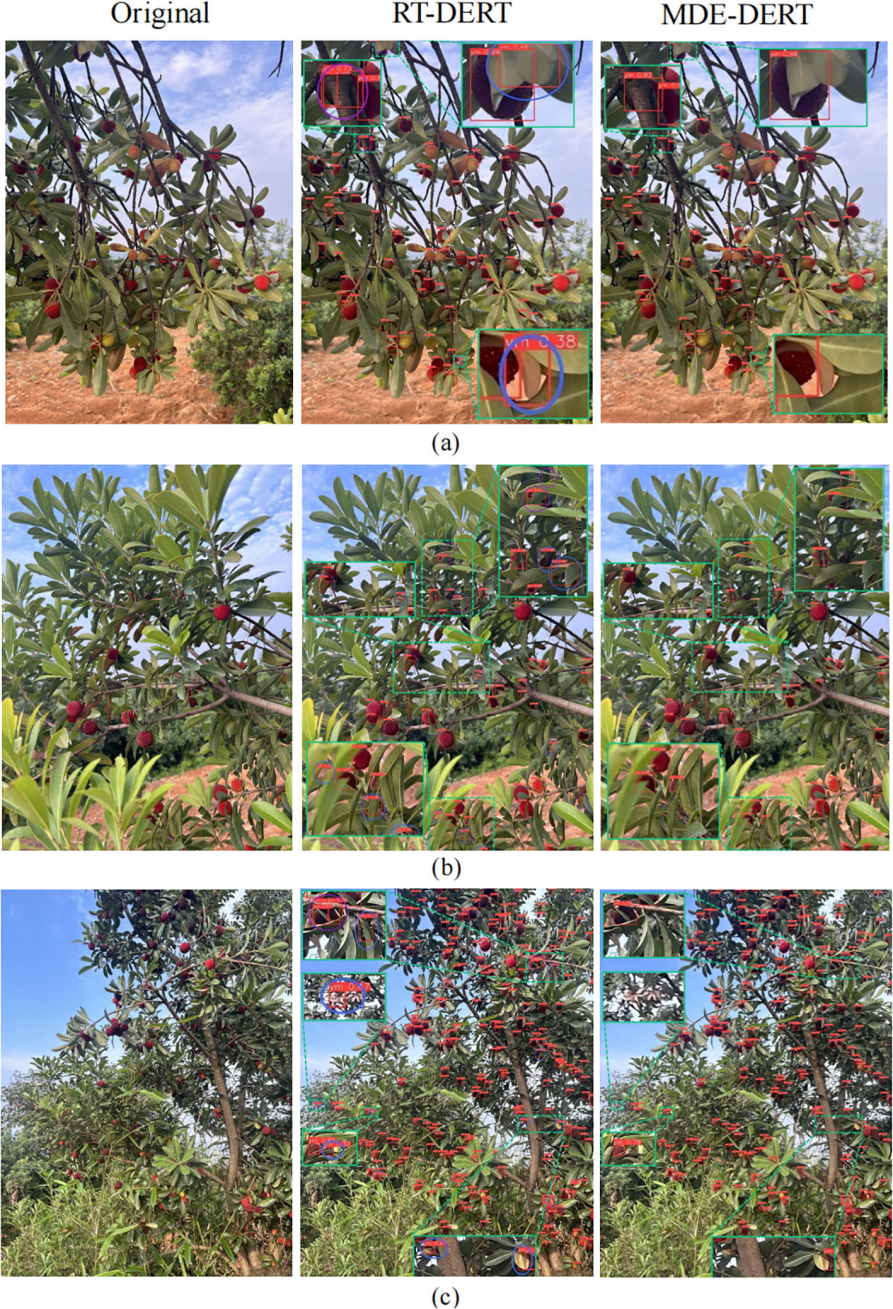
Partial image detection results. **(a)** dense distribution; **(b)** leaf occlusion; **(c)** small targets.

To intuitively demonstrate improvement effects, this study selected representative images from three typical complex scenarios: dense distribution, leaf occlusion, and small targets, for visual comparison analysis between the RT-DETR baseline model and the improved MDE-DETR model. As shown in **Figure 10**, differentiated color marking is used to highlight key performance comparisons: blue regions identify false detection phenomena in the RT-DETR model (non-bayberry targets incorrectly identified), and purple regions identify missed detection or duplicate detection phenomena (target omission or multiple identifications of the same target). Comparison results show that MDE-DETR effectively improved detection performance through synergistic effects of three core innovation modules, demonstrating significant advantages in target localization accuracy, detection confidence, and completeness.

Specific scenario analysis shows: In dense distribution scenarios (**Figure 10a**), RT-DETR tends to miss detections in target overlap areas due to limitations in feature extraction and fusion capabilities. MDE-DETR effectively improved dense target detection accuracy through multi-path feature enhancement mechanisms in the EFENet backbone’s MFEM module, combined with SPDConv spatial pixel rearrangement and CMKBlock omnidirectional kernel feature enhancement strategies in MDFFN. In occlusion scenarios (**Figure 10b**), RT-DETR’s fixed sampling mechanism struggles to adapt to complex morphologies of occluded targets. MDE-DETR employs the ADSample adaptive deformable downsampling module, significantly enhancing detection robustness in occluded environments through learnable spatial offset prediction mechanisms that dynamically adjust sampling positions. In small target detection scenarios (**Figure 10c**), RT-DETR has insufficient utilization of multi-scale feature information, with limited accuracy when processing distant small-size targets. MDE-DETR significantly improved small target feature expression and recognition accuracy through efficient feature extraction capabilities of reparameterized convolution technology in EFENet, combined with spatial-frequency domain synergistic optimization of DDAM dual-domain attention mechanisms in MDFFN.

#### 4.3.5 Model feature visualization

**Figure 11:**
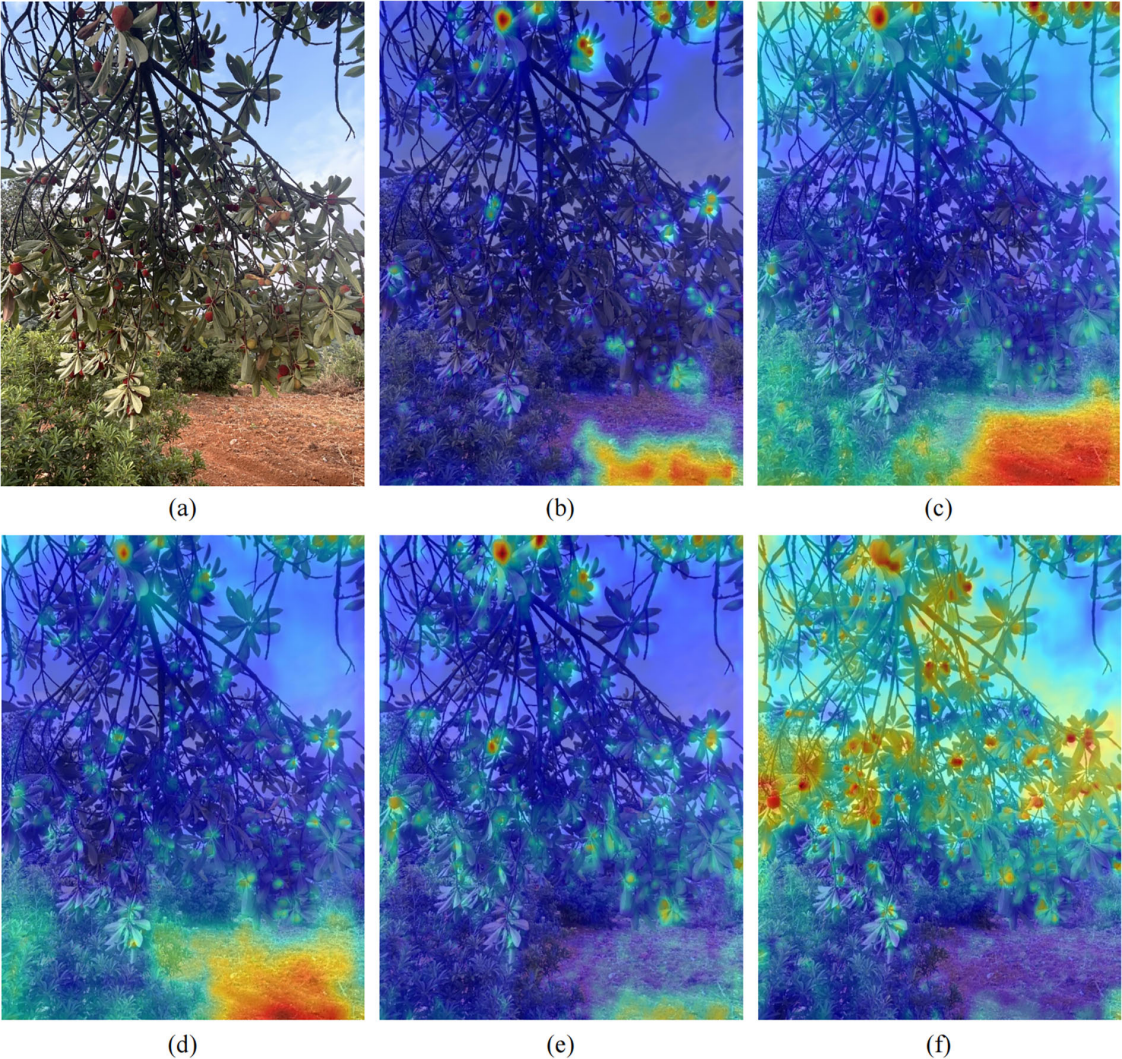
Heatmap visualization results of different models. **(a)** Original; **(b)** RTDETR-r18 model; **(c)** RTDETR-r34 model; **(d)** RTDETR-r50 model; **(e)** yolo12m model; **(f)** MDE-DETR model.

To more intuitively observe the improvement in MDE-DETR model’s bayberry recognition capability, this study employed Grad-CAM (Gradient-weighted Class Activation Mapping) technology to generate heatmaps, visually demonstrating the model’s learning of bayberry features. Grad-CAM utilizes training weights for backpropagation, performs global average pooling on gradient matrices in spatial dimensions, and conducts weighted activation processing on various channels of feature layers to generate visualization heatmaps. In these heatmaps, regional brightness directly reveals feature areas with significant impact on model prediction results. The heatmap visualization comparison shown in **Figure 11** clearly demonstrates performance differences among different models. Compared to current mainstream models RT-DETR-r18, RT-DETR-r34, RT-DETR-r50, and YOLO12m, the MDE-DETR model shows brighter colors in bayberry target regions, indicating significantly enhanced response to bayberry features. Particularly in the MDE-DETR model heatmap (f) in the bottom right of the image, bayberry regions show distinct yellow-red highlighted areas, indicating that the model can more accurately focus on key features in bayberry regions.

#### 4.3.6 Generalization experiments

To verify the generalization performance of the proposed method, this study conducted comparative experiments between MDE-DETR and RT-DETR-r18 on the VisDrone2019 (Du et al., 2019) drone aerial small target dataset and TomatoPlantfactoryDataset (Wu et al., 2023) tomato dataset. as shown in the **Table 5**.The rationale for selecting these two datasets is that VisDrone2019 dataset primarily features small targets, while TomatoPlantfactoryDataset has characteristics such as dense occlusion, diverse fruit morphologies, and complex lighting conditions, which share high similarity with our bayberry dataset. Experimental environment and parameter configurations remained consistent with Section 4.1. Experimental results show that on the VisDrone2019 dataset, compared to RT-DETR-r18, MDE-DETR improved precision and recall by 1.8% and 4.0% respectively, significantly reducing missed and false detections of small targets in drone aerial scenarios; meanwhile, mAP50 and mAP50:95 improved by 3.0% and 1.6% respectively, indicating superior detection accuracy of MDE-DETR. On the TomatoPlantfactoryDataset dataset, MDE-DETR’s precision and recall improved by 1.9% and 1.3% respectively compared to RT-DETR-r18, effectively improving tomato detection performance in complex orchard environments; mAP50 and mAP50:95 improvements reached 1.8 and 1.5 percentage points respectively, further confirming MDE-DETR’s superior performance. Results show that the proposed MDE-DETR model demonstrates superior detection performance on two datasets with different characteristics, fully validating the method’s good generalization capability.

**Table 5:** Generalization experiments on different datasets.

Datasets	Model	P(%)	R(%)	mAP50(%)	mAP50:95(%)
VisDrone2019	RT-DERT-r18	61.9	46.1	47.5	29.1
	MDE-DETR	63.7	50.1	50.5	30.7
TomatoPlantfactoryDataset	RT-DERT-r18	89.1	83.5	88.7	68.5
	MDE-DETR	91.0	84.8	90.5	70.0

#### 4.3.7 Bayberry detection and counting correlation study

**Figure 12:**
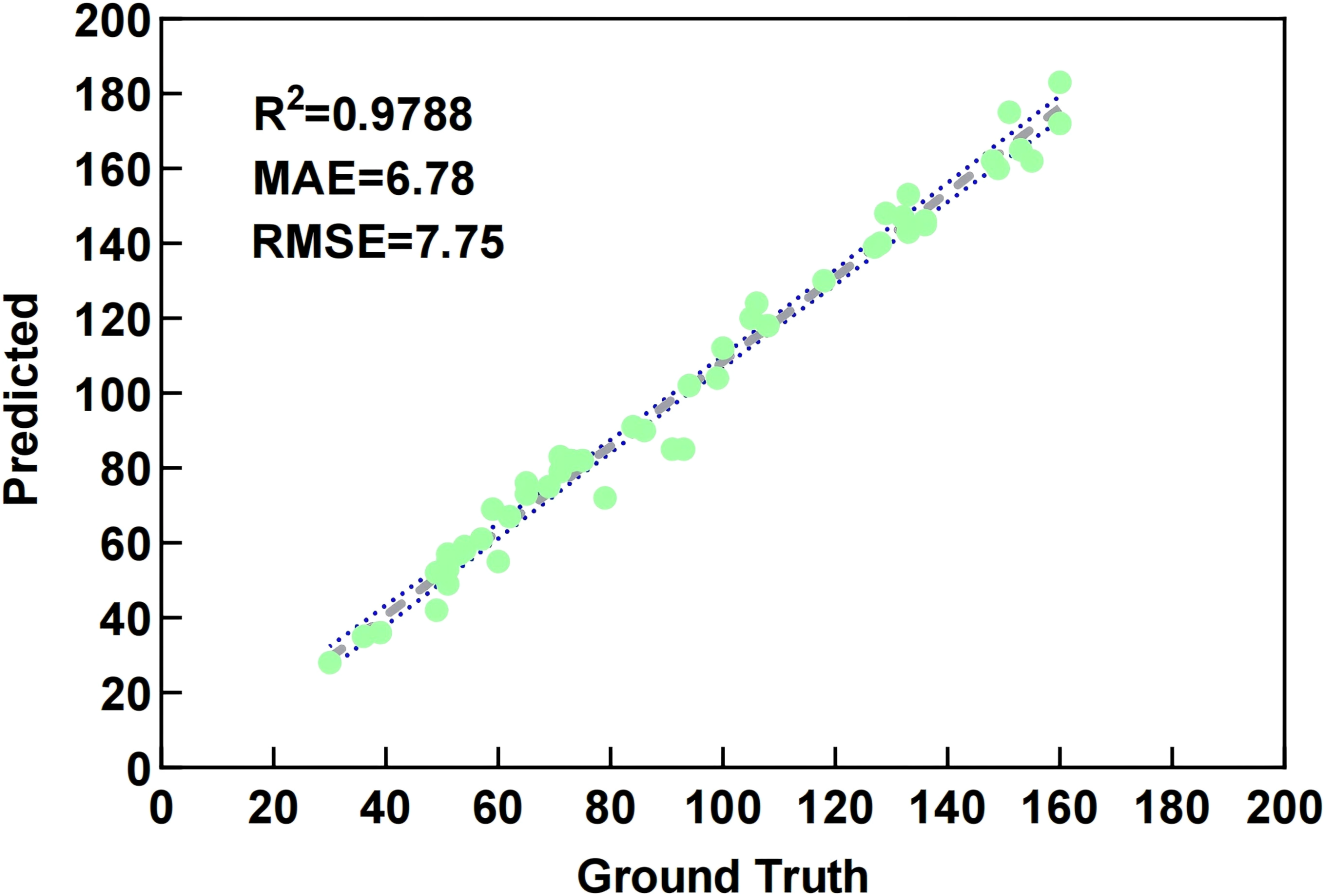
Linear regression analysis comparison between manual counting and machine counting.

To verify the effectiveness of the proposed bayberry detection and counting method, this study randomly selected 50 images from the test set for validation. To ensure accuracy of standard data, bayberries in each image were manually counted 5 times, with average values taken as ground truth annotations. Linear regression analysis was used to quantitatively evaluate MDE-DETR model’s detection performance. As shown in **Figure 12**, the fitting curve between machine counting and manual counting results shows good linearity, with R² = 0.9788 indicating high linear correlation. The Mean Absolute Error (MAE = 6.78) shows that on average, the model’s predictions deviate from manual counts by approximately 6.78 bayberries, demonstrating good practical accuracy. The Root Mean Square Error (RMSE = 7.75) confirms the model’s consistency across the test set. These comprehensive metrics validate the feasibility and accuracy of MDE-DETR model for precise bayberry counting.

**Figure 13:**
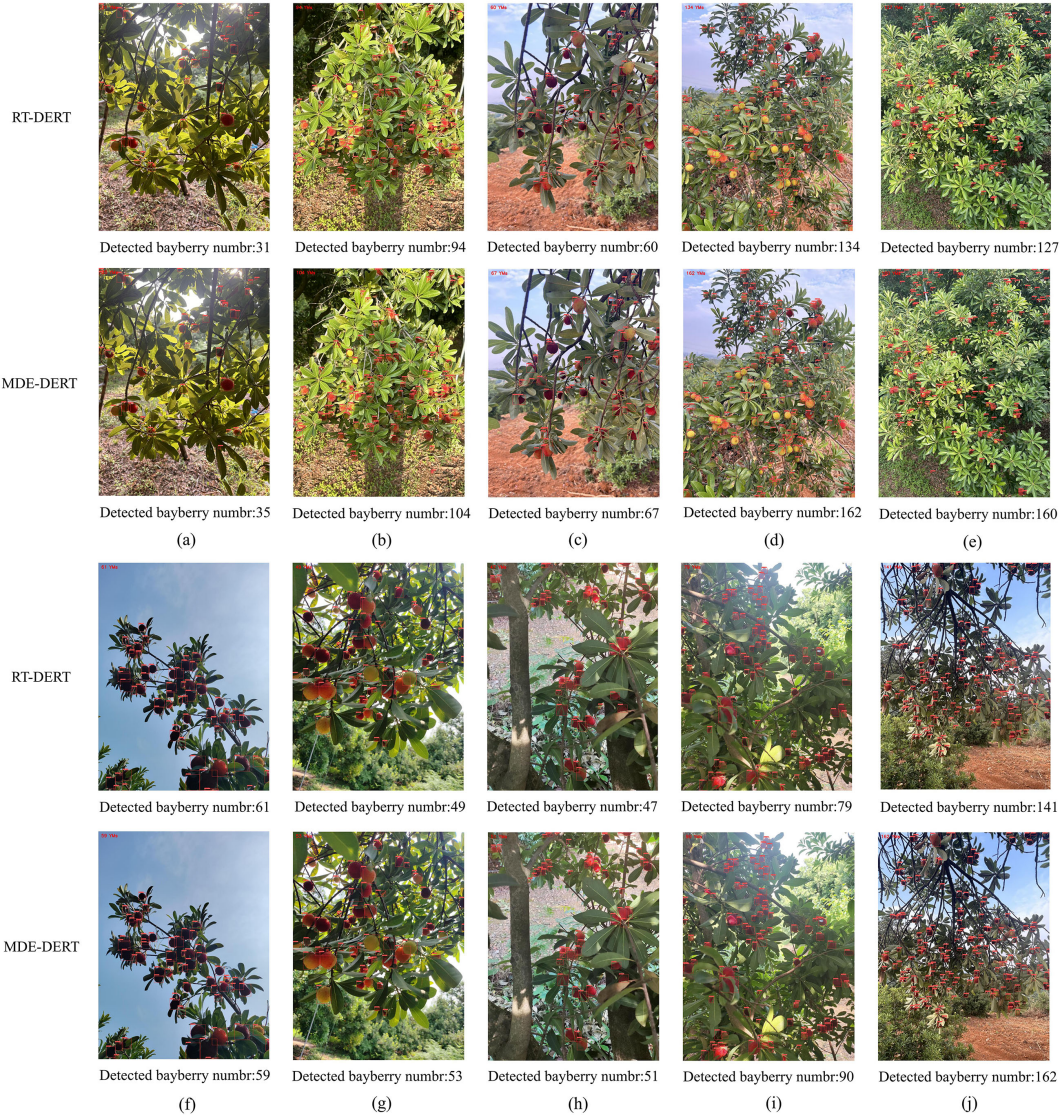
Partial image detection and counting results. **(a)** Back light; **(b)** Direct light; **(c)** Close shot; **(d)** Medium shot; **(e)** Long shot; **(f)** Weak light; **(g)** Mixed bayberry; **(h)** Red fruit; **(i)** Dense; **(j)** Severe occlusion.

**Figure 13** shows bayberry detection and counting results of the MDE-DETR model in various complex scenarios. Experimental results demonstrate that bayberry quantities calculated by the model highly match actual manual counts, further validating the reliability of the research method. Through visual comparison analysis on the test set, it was found that in various complex scenarios including occlusion, dense distribution, close-up, medium, and distant views, the baseline model RT-DETR’s detection accuracy was significantly lower than the improved MDE-DETR model, with performance differences being more pronounced especially in challenging scenarios such as medium-distant small targets, severe occlusion, and high-density distribution.

## 5 Discussion

The MDE-DETR algorithm proposed in this study achieved significant success in bayberry detection tasks in complex orchard environments, demonstrating clear advantages over existing methods. First, addressing the characteristics of dense distribution and easy occlusion of small bayberry targets, traditional YOLO series algorithms performed poorly, while this study’s RT-DETR architecture achieved end-to-end object detection, eliminating post-processing steps such as NMS, simplifying the detection process and improving efficiency. Compared to Li et al.’s improved YOLOv7 bayberry detection model that achieved high accuracy of 90.21% but with parameter count as high as 124.5M (Li et al., 2023), this study achieved 92.9% detection accuracy with only 14.7M parameters, making it more suitable for mobile and embedded device deployment requirements.

In terms of technical innovation, the EFENet backbone network effectively addressed the problem of insufficient feature expression capability of traditional ResNet in complex environments through multi-path feature enhancement via MFEM modules. The MDFFN feature fusion architecture significantly improved small target detection accuracy through lossless dimensional transformation of SPDConv modules and omnidirectional kernel feature enhancement of CMKBlock. The adaptive offset learning mechanism of ADSample modules enabled downsampling processes to dynamically adapt to target geometric features, performing particularly well in occlusion and dense scenarios.

Generalization experiments on VisDrone2019 drone aerial small target dataset and TomatoPlantfactoryDataset tomato dataset demonstrated that the MDE-DETR model has good cross-domain adaptation capability.

While our dataset of 1,954 high-quality images was collected under diverse conditions (different lighting, angles, and growth stages), it originates from a single geographical location. This may limit the model’s generalization to bayberry varieties, cultivation practices, or environmental conditions found in other regions. The dataset primarily captures the Dongkui bayberry variety common to Zhejiang Province, and performance may vary with other varieties.

To partially mitigate these concerns, we conducted generalization experiments on two additional datasets: VisDrone2019 (a drone aerial small target dataset) and TomatoPlantfactoryDataset (a dense occlusion dataset). The strong performance on these diverse datasets suggests reasonable generalization capability to similar small-target detection tasks. However, we acknowledge that validation on additional bayberry datasets from different geographical regions would further strengthen confidence in the model’s robustness.

However, this study still has certain limitations: although the model has optimizations in parameter count, processing of P2 layer in MDFFN modules still brings certain computational overhead; the dataset scale is relatively limited, and larger-scale, more diverse datasets might further improve model performance.

Future work will focus on: (1) collecting multi-regional bayberry datasets covering different varieties and cultivation environments, (2) investigating domain adaptation techniques to improve cross-regional performance, and (3) validating the model on commercial orchards in different geographical locations.

## 6 Conclusion

This paper proposes an MDE-DETR algorithm for small-target bayberry detection and counting in complex orchard environments. By constructing EFENet enhanced feature extraction network, MDFFN multi-domain feature fusion architecture, and ADSample adaptive deformable downsampling module, technical challenges of small target detection, dense distribution, and frequent occlusion were successfully addressed. Experimental results show that compared to the RT-DETR baseline model, MDE-DETR achieved improvements of 3.8% and 5.1% in mAP50 and mAP50:95 indicators respectively, reaching detection accuracies of 92.9% and 67.9%, while reducing parameters and memory usage by 25.76% and 25.14% respectively, achieving a good balance between detection accuracy and computational efficiency. Generalization experiments further validated the robustness and applicability of the method. This algorithm provides an efficient and feasible technical solution for fruit detection and counting in precision agriculture, with important theoretical value and practical significance. Future work will focus on further optimizing model structure, expanding dataset scale, and exploring application potential in broader agricultural scenarios.

## Data Availability

The raw data supporting the conclusions of this article will be made available by the authors, without undue reservation.
